# Size-Dependent High-Pressure Behavior of Pure and Eu^3+^-Doped Y_2_O_3_ Nanoparticles: Insights from Experimental and Theoretical Investigations

**DOI:** 10.3390/nano14080721

**Published:** 2024-04-20

**Authors:** André Luis de Jesus Pereira, Juan Ángel Sans, Óscar Gomis, David Santamaría-Pérez, Sudeshna Ray, Armstrong Godoy, Argemiro Soares da Silva-Sobrinho, Plácida Rodríguez-Hernández, Alfonso Muñoz, Catalin Popescu, Francisco Javier Manjón

**Affiliations:** 1 Instituto de Diseño para la Fabricación y Producción Automatizada, MALTA Consolider Team, Universitat Politècnica de València, 46022 València, Spain; juasant2@upvnet.upv.es; 2 Laboratório de Plasmas e Processos—LPP, Instituto Tecnológico de Aeronáutica—ITA, São José dos Campos 12228-900, Brazil; godoyajr@gmail.com (A.G.J.); argemirosss@gmail.com (A.S.d.S.-S.); 3 Centro de Tecnologías Físicas, MALTA Consolider Team, Universitat Politècnica de València, 46022 València, Spain; osgohi@fis.upv.es; 4 Departament de Física Aplicada-ICMUV, MALTA Consolider Team, Universitat de Valencia, 46100 Burjassot, Spain; david.santamaria@uv.es; 5 Department of Chemistry, Rabindranath Tagore University, Bhopal 464993, Madhya Pradesh, India; sudeshnaskype@gmail.com; 6 Departamento de Física, Instituto de Materiales y Nanotecnología, MALTA Consolider Team, Universidad de La Laguna, 38207 San Cristóbal de La Laguna, Spain; plrguez@ull.edu.es (P.R.-H.); amunoz@ull.edu.es (A.M.); 7 ALBA-CELLS, MALTA Consolider Team, 08290 Cerdanyola del Valles (Barcelona), Catalonia, Spain; cpopescu@cells.es

**Keywords:** high-pressure, nanoparticles, Eu-doped, yttrium oxide, pressure, induced amorphization

## Abstract

We report a joint high-pressure experimental and theoretical study of the structural, vibrational, and photoluminescent properties of pure and Eu^3+^-doped cubic Y_2_O_3_ nanoparticles with two very different average particle sizes. We compare the results of synchrotron X-ray diffraction, Raman scattering, and photoluminescence measurements in nanoparticles with ab initio density-functional simulations in bulk material with the aim to understand the influence of the average particle size on the properties of pure and doped Y_2_O_3_ nanoparticles under compression. We observe that the high-pressure phase behavior of Y_2_O_3_ nanoparticles depends on the average particle size, but in a different way to that previously reported. Nanoparticles with an average particle size of ~37 nm show the same pressure-induced phase transition sequence on upstroke and downstroke as the bulk sample; however, nanoparticles with an average particle size of ~6 nm undergo an irreversible pressure-induced amorphization above 16 GPa that is completed above 24 GPa. On downstroke, 6 nm nanoparticles likely consist of an amorphous phase.

## 1. Introduction

The search for continuous technological innovation means that many efforts are directed toward the development and in-depth understanding of the properties of new functional materials. In this respect, lanthanide sesquioxides with formula Ln_2_O_3_ (Ln = La to Lu, Y, and Sc) have stood out for their unique and interesting properties, especially when thinking about optical, catalytic, and electronic applications. Among Ln_2_O_3_ compounds, yttrium oxide (Y_2_O_3_) has garnered significant attention in scientific and technological circles owing to its non-toxicity, biocompatibility, and natural abundance. This interest scales when Y_2_O_3_ nanoparticles are doped with other rare-earth (RE) elements, such as Tm, Er, and Eu, since RE-doped Y_2_O_3_ is considered an excellent optical material for IR windows, phosphors, and solid-state lasers due to its high melting point, hardness, chemical stability, and good optical properties [1]. Y_2_O_3_ stands out not only for its distinctive properties among Ln_2_O_3_ compounds but also for its notable ease and stability in accommodating the doping of various RE atoms [2]. This characteristic makes Y_2_O_3_ a potential candidate for red emitters for solid-state lighting applications, bio imaging, and anti-counterfeiting technologies [2].

The intrigue stems from pronounced and well-defined electronic transitions linked to electrons at the 4f levels of RE trivalent ions nestled within the Y_2_O_3_ lattice. These transitions give rise to robust and enduring emissions in specific regions of the electromagnetic spectrum, critical for diverse optoelectronic applications, including high-density optical storage and electroluminescent devices. In the context of Eu^3+^-doped Y_2_O_3_ (Y_2_O_3_:Eu^3+^), the morphology, particle size, crystalline structure, and dopant concentration directly influence its luminescent properties [3,4,5,6,7,8,9].

The ground state configuration of Ln atoms in RE sesquioxides (RE-SOs) is predominantly Ln^3+^, with the notable exceptions of Ce and Tb. While Ce typically exhibits the tetravalent Ce^4+^ state, Tb deviates from the common Ln^3+^ configuration and naturally appears as Tb_4_O_7_, presenting Tb atoms in both 3+ and 4+ valence states [10,11]. It is worth noting that Pr is another rare case, exhibiting a mixed 3+/4+ oxidation state in oxides [12]. Under ambient conditions, RE-SOs exhibit mainly three structural modifications named C-type (space group (S.G.) Ia-3, No. 206, Z = 16), B-type (S.G. C2/m, No. 12, Z = 6), and A-type (S.G. $P\bar{3}m1$, No. 164, Z = 1). The cubic phase has a bixbyite structure that contains 16 formula units of Ln_2_O_3_ with T_h_^7^ symmetry and the resulting structure (with 32 Ln^3+^ ions) has 24 Ln^3+^ ions on sites with C_2_ symmetry and 8 Ln^3+^ ions on sites with C_3i_ (or S_6_) symmetry [13,14]. The monoclinic phase contains six formula units of Ln_2_O_3_ with C_2h_^3^ symmetry and the resulting structure (with 12 Ln^3+^ ions) has the 12 Ln^3+^ ions on three different 4*i* sites with C_s_ symmetry [15]. Finally, the trigonal A-type phase contains only one formula unit of Ln_2_O_3_ with D_3d_^3^ symmetry and the resulting structure (with two Ln^3+^ ions) has the two Ln^3+^ ions on 2*d* sites with C_3v_ symmetry [16].

As nanostructures, these RE-SOs emerge as promising materials, thanks to the increase in the surface area and the effects of quantum confinement [17]. The nanosized versions of these RE-SOs exhibit considerable potential across a diverse array of applications. These applications include, but are not limited to, fuel cells, solid-state light-emitting devices, luminescent probes in immunoassays, chemical−mechanical polishing (CMP), ultra-fine polishing, catalytic converters, catalysis, high-efficiency luminescent materials (for flat panel displays, plasma displays), red powder activation of color TV, sintering aids, RE magnets, control rod material for fast breeder reactors, and more [17]. Although some studies have focused on investigating the properties of Y_2_O_3_:Eu^3+^ nanoparticles, there is still a scarcity of systematic works that integrate both experimental and theoretical results, enabling a more detailed understanding of the effects of pressure on their properties.

In a previous work, we characterized at room pressure the structural, vibrational, and photoluminescent properties of pure and Eu^3+^-doped (1 at. wt%) Y_2_O_3_ nanoparticles with different sizes [18]. Now, we report a joint experimental and theoretical investigation of the structural, vibrational, and photoluminescence properties of pure and Eu^3+^-doped (1 at. wt%) Y_2_O_3_ nanoparticles with two very different average sizes (6 nm and 37 nm) at high pressure (HP) using a multi-technique approach by means of X-ray diffraction (HP-XRD), Raman scattering (HP-RS), and photoluminescence (HP-PL) measurements. A special emphasis is placed on understanding the influence of the average nanoparticle size on their HP properties. For this purpose, we analyzed all our experimental results in light of previous experimental and theoretical results for bulk and nanosized Y_2_O_3_ at HP. Our measurements in pure and doped Y_2_O_3_ nanoparticles evidence that the behavior of nanoparticles under compression depends on the average particle size. Nanoparticles with an average particle size of ~37 nm show the same pressure-induced phase transition (PT) sequence (C-B-A) on upstroke and (A-B) downstroke as the bulk sample [19]. This is in contrast with what has been published in many previous works [20,21,22,23,24,25]. On the other hand, nanoparticles with an average particle size of ~6 nm undergo an irreversible pressure-induced amorphization (PIA) starting above 16 GPa, with full amorphization observed above 24 GPa.

## 2. Materials and Methods

### 2.1. Experimental Procedure

The nanocrystalline samples studied in this work were synthetized by a complex-based precursor solution method. All details regarding the synthesis and ambient pressure characterization procedure can be found elsewhere [18]. Briefly, Y_2_O_3_ nanoparticles, both undoped and Eu^3+^-doped, were synthesized using a complex-based precursor solution method with triethanolamine (TEA) as a complexing agent. A clear solution of TEA-complexed metal nitrate was evaporated to form a voluminous organic-based precursor powder. This powder underwent calcination at 500 °C and 1000 °C for 2 h [18]. High-resolution transmission electron microscopy (HRTEM) measurements were utilized to examine the morphology and dimensions of yttria nanoparticles, both doped with Eu^3+^ and undoped. These nanoparticles exhibited predominantly spherical shapes. Upon annealing at 500 °C and 1000 °C for 2 h, the average sizes were determined to be 6 nm and 37 nm, respectively, based on measurements of over 100 particles [18]. Notably, the size distribution showed a variation of less than ±15% from the average size. These nanoparticle samples are hereafter noted as Y_2_O_3_-6 nm, Y_2_O_3_:Eu^3+^-6 nm, Y_2_O_3_-37 nm, and Y_2_O_3_:Eu^3+^-37 nm.

Powder angle-dispersive HP-XRD measurements were performed at room temperature at the BL04-MSPD beamline of the ALBA-CELLS synchrotron [26] using a monochromatic X-ray beam with λ = 0.4246 Å. The sample was loaded in a Merrill–Bassett-type diamond anvil cell (DAC) with diamond culets of 400 μm in diameter together with a 16:3:1 methanol–ethanol–water mixture as a pressure-transmitting medium (PTM) [27] and pressure was estimated from the equation of state (EoS) of copper [28]. The Dioptas software [29] was used to integrate 2D diffraction images. Finally, structural analysis through Rietveld and Le Bail refinements were performed using FullProf [30] and PowderCell [31] program packages.

Unpolarized RS and PL measurements excited with a 532 nm laser with a power of less than 10 mW were taken in backscattering geometry using a Horiba Jobin Yvon LabRAM HR UV microspectrometer equipped with a thermoelectrically cooled multichannel charge-coupled device detector and a 1200 grooves/mm grating that allows a spectral resolution better than 3 cm^−1^. The sample was loaded in a membrane-type DAC with a 16:3:1 methanol–ethanol–water mixture and pressure was determined by the ruby luminescence method [32]. Raman peaks were analyzed with a Voigt profile fixing the Gaussian linewidth (2.4 cm^−1^) to the experimental setup resolution. In all experiments, the DAC loading was performed taking care of avoiding sample bridging between the diamonds [33].

### 2.2. Simulation Details

Ab initio total-energy calculations at 0 K for the C-, B-, and A-type phases of bulk Y_2_O_3_ were performed within the Density Functional Theory (DFT) [34] framework with the Vienna Ab-initio Simulation Package (VASP) [35,36], as already reported in ref. [19]. The pseudopotential method and the projector augmented waves (PAW) scheme [37,38] were used with the plane-wave basis set extended up to an energy cutoff of 520 eV. The generalized gradient approximation (GGA), with the Perdew–Burke–Ernzerhof parametrization extended for solids (PBEsol) [39], was used to describe the exchange and correlation energy. The Brillouin zones of these structures were sampled with dense Monkhorst–Pack meshes [40] of special *k*-points (6 × 6 × 6 for the C-type phase, 7 × 7 × 3 for the B-type phase, and 6 × 6 × 6 for the A-type phase, using the primitive cell). This method ensures a high convergence of 1–2 meV per formula unit in the total energy and an accurate calculation of the forces on atoms. For each of the studied phases, the structures were fully relaxed to the optimized configuration, at sets of selected volumes, through the calculation of the forces on atoms and the stress tensor. Two optimization criteria were used: (i) forces on the atoms should be lower than 0.005 eV/Å and (ii) deviations of the stress tensor from the diagonal hydrostatic form should be lower than 0.1 GPa. The direct-force constant approach [41] was used to obtain lattice-dynamical properties, frequency, and symmetry of the phonon modes at the Γ point of the Brillouin zone.

## 3. Results and Discussion

As previously commented, the nanoparticles studied in this work at high pressure were fully characterized at room pressure in a previous work, where a comparison of the structural, vibrational, and photoluminescent properties of the pure and Eu^3+^-doped Y_2_O_3_ nanoparticles was reported [18]. In that work, photoluminescent properties were compared only for doped nanocrystals, while structural and vibrational properties were compared among all doped and undoped nanocrystals. Structural properties showed no difference between pure and doped nanocrystals of the same average size; however, vibrational properties of pure and doped nanocrystals of the same average size were clearly different (see Figure S1). Our pure Y_2_O_3_ nanoparticles of 37 nm and 6 nm have similar RS spectra to those already reported for bulk Y_2_O_3_ [13,42,43,44,45,46], and nanocrystalline Y_2_O_3_ samples [20,25,47,48,49], presenting only a significant difference related to the width of the peaks. The RS spectra are dominated by an intense peak around 377 cm^−1^ and eight less intense peaks, consistent with the spectra presented by other studies. On the other hand, Eu^3+^-doped Y_2_O_3_ nanoparticles of 37 nm and 6 nm show different RS spectra. The RS spectrum of the Eu^3+^-doped nanoparticles of 37 nm is similar to those of pure nanoparticles; however, the RS spectrum of Eu^3+^-doped nanoparticles of 6 nm does not show most of the Raman peaks of C-type Y_2_O_3_, except the most intense 377 cm^−1^ peak. In addition, the RS spectra of Eu^3+^-doped nanoparticles present four new peaks at 429, 450, 489, and 603.6 cm^−1^ that are not present in the undoped samples (peaks marked with asterisks in Figure S1). They can be assigned to PL peaks of the Eu^3+^ ion in the Y_2_O_3_ matrix. At present, it is not fully clear whether they correspond to the ^5^D_0_-^7^F_3_ transition of Eu^3+^ ions that appears at similar frequencies [18,50,51,52]. In any case, similar RS spectra have been measured in Y_2_O_3_:Eu^3+^ nanotubes excited with 514.5 nm [22]. As a consequence of the observation of these PL peaks, RS measurements in Eu^3+^-doped nanoparticles do not show most of the Raman-active modes expected for cubic Y_2_O_3_ and its study can be confusing (see Figures S2 and S3). Consequently, we will show in the following HP-XRD and HP-RS measurements for pure nanoparticles and HP-PL measurements for doped nanoparticles in order to characterize the structural, vibrational, and photoluminescent properties of the yttria nanocrystals.

### 3.1. Y_2_O_3_-37 nm and Y_2_O_3_:Eu^3+^-37 nm Nanocrystals

**Figure 1:**
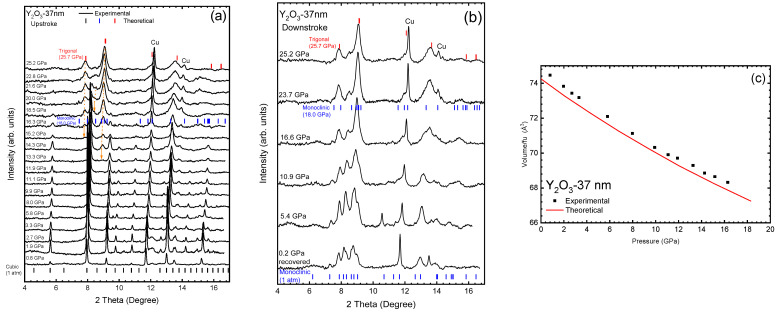
Room-temperature XRD patterns of Y_2_O_3_-37 nm at selected pressures on (**a**) upstroke and (**b**) downstroke. The orange arrows presented in (**a**) show the new peaks that appear with the pressure increase. Black, red and blue symbols in figure (**a**,**b**) are the position of the diffraction peaks of the C-, A-, and B-type phases, respectively, according to the data obtained from our theoretical calculation. (**c**) Pressure dependence of the experimental (Y_2_O_3_-37 nm, black symbols) and theoretical (red line) unit-cell volume per formula unit of the cubic phase on upstroke.

In order to undertake the structural characterization of the Y_2_O_3_-37 nm nanocrystals, we performed synchrotron-based HP-XRD measurements (Figure 1). A comparison of Figure 1a,b with HP-XRD measurements of the bulk Y_2_O_3_ [19] evidences a very similar behavior of Y_2_O_3_-37 nm and Y_2_O_3_-Bulk samples. As pressure increases the peaks of the C-type phase in Y_2_O_3_-37 nm tend to displace to higher angles, indicating a decrease in the unit-cell volume. Above 13.3 GPa, the peaks related to the C-type phase start to broaden and loose intensity, while new peaks appear at ~8.9° (13.3 GPa) and 7.8° (15.2 GPa) (see orange arrows in Figure 1a). As in Y_2_O_3_-Bulk, these new peaks can be respectively related to planes $\left(40\bar{2}\right)$ and (111) of the B-type Y_2_O_3_ or to planes (101) and (100) of the A-type Y_2_O_3_. Additionally, a small peak appears at 18.5 GPa at ~8.4° that corresponds to the diffraction of the (401) plane of the B-type phase. The main peak of the cubic phase disappears in Y_2_O_3_-37 nm above 21.6 GPa in good agreement with the Y_2_O_3_-Bulk that disappears above 22 GPa. Furthermore, at 25.2 GPa we observed a broad peak at ~13.6° which may contain a contribution that can be related to the A-type Y_2_O_3_ (003) plane. Thus, as mentioned for Y_2_O_3_-Bulk, the peaks that appear at 13.3, 15.2, and 18.5 GPa may initially belong to the B-type phase, indicating a mixture between the B- and C-type phases. At even higher pressures there is a PT to the A-type phase in Y_2_O_3_-37 nm as in Y_2_O_3_-Bulk [19]. These results regarding the pressure-induced C-B-A PT sequence in Y_2_O_3_-37 nm on increasing pressure are further confirmed by our RS measurements (later commented).

As regards downstroke, a clear A-B PT occurs already at 16.6 GPa on decreasing pressure with the appearance of new diffraction peaks that remain in the recovered sample at 0.2 GPa. In particular, new diffraction peaks appear near 7.2°, 11.2°, and 13.2° at 16.6 GPa that clearly correspond to the B-type phase. Therefore, we can also conclude that Y_2_O_3_-37 nm behaves as in Y_2_O_3_-Bulk on decreasing pressure.

The quality of the HP diffractograms only allowed us to perform Le Bail refinements up to 12 GPa for the cubic phase of Y_2_O_3_. Above this pressure, diffractograms are not sufficiently defined to obtain a reliable refinement. As for the bulk sample, we have observed a good agreement between the experimental and theoretical (bulk) pressure dependence of the unit-cell volume (see Figure 1c). The 3^rd^-order BM-EoS fit for Y_2_O_3_-37 nm results in V_0_/Z = 74.82(5) Å^3^, B_0_ = 144(7) GPa, and B_0_′ = 3(1). The modified BM-EOS fit also resulted in an axial compressibility of κ_a_ = 2.5(5) × 10^−3^ GPa^−1^ (Table 1). The B_0_ value compares well with previous reported values in nanotubes (131–145 GPa) [22,23,25]. As can be seen in Table 1, these values are very similar to those obtained for both the bulk sample and the theoretical calculation, indicating that Y_2_O_3_-37 nm nanocrystals have a similar behavior as the bulk sample at HP [19]. These results support the conclusions of the study of Wang et al. that observed a bulk behavior In nanoparticles up to 21 nm [20].

**Figure 2:**
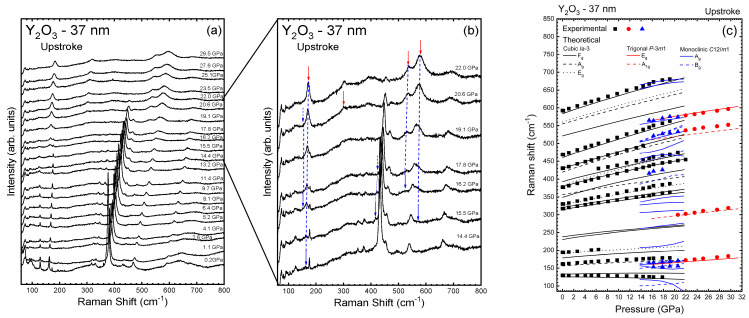
(**a**) Room-temperature Raman spectra of Y_2_O_3_-37 nm at selected pressures on upstroke. (**b**) Details of the measurements carried out between 14.4 GPa and 22.0 GPa (on upstroke), where it is possible to detect five new peaks indicated by blue arrows. Dashed blue arrows indicate the last pressure that two of these peaks are observed. (**c**) Pressure dependence of the Raman-active modes of Y_2_O_3_-37 nm on upstroke. Black, blue, and red symbols and lines represent the experimental and theoretical data for the C-, B-, and A-type phases, respectively.

Figure 2a shows the RS spectra of pure Y_2_O_3_-37 nm at selected pressures up to 29.5 GPa. In the RS spectrum of Y_2_O_3_-37 nm we have been able to follow the same nine Raman-active modes already observed in C-type bulk Y_2_O_3_ and with a similar pressure dependence as in bulk Y_2_O_3_ [19] (Figure 2c). The good agreement between experimental and theoretical results could be confirmed by the mode of the cubic phase initially at 392.7 cm^−1^ that is not detected at low pressures but that is well observed above 6 GPa (Figure 2a). Based on its pressure evolution, we believe that this peak can be identified as the *F_g_*^10^ mode (see Table 2) of the C-type phase and that, at low pressure, was overlaid by the most intense peak (initially at 377.2 cm^−1^). Table 2 summarizes the zero-pressure frequencies and their pressure coefficients for the experimental Raman-active modes of the Y_2_O_3_-37 nm sample. The experimental results for the Y_2_O_3_-37 nm sample are in good agreement with the theoretical results for bulk Y_2_O_3_, which allows us to make a direct comparison between the two results with a relatively high degree of confidence.

Up to 11.4 GPa, only the peaks of the cubic phase are observed, with no PT or mixture of phases being evident. At 14.4 GPa some changes occur in the RS spectrum. Figure 2b shows selected RS spectra between 14.4 and 22.0 GPa where new peaks related to B- and A-type phases are observed in the upstroke and marked by blue and red arrows, respectively. In particular, the *F_g_*^1^ mode of the cubic phase (at ~130 cm^−1^) suffers a considerable broadening and a new peak around 160 cm^−1^ appears (see blue arrow in Figure 2b). These two changes are followed by the appearance of two broad bands between 500 and 600 cm^−1^ at 16.2 GPa and a broad band without clear maxima between 200 and 350 cm^−1^ (see blue arrows in Figure 2b). Additionally, a shoulder near 150 cm^−1^ and a weak peak at 420 cm^−1^ are observed above 16.2 and 15.5 GPa, respectively (see blue arrows in Figure 2b). These modes can be assigned to the B-type phase. Therefore, our detailed study indicates that there is a C-B PT around 14.4 GPa (above this pressure there is a considerable decrease in the intensity of the cubic *F_g_*^10^ mode), with coexistence of both cubic and monoclinic phases up to 21.9 GPa. This result contrasts with previous studies on Y_2_O_3_ nanocrystals that showed no signal of the B-type phase on upstroke [20].

A new peak around 300 cm^−1^ appears in the 200–350 cm^−1^ broad band at 20.6 GPa (see red arrow in Figure 2b). This new peak indicates that the C-B PT is followed by the emergence of the A-type phase above this pressure. At 22.0 GPa, the four peaks corresponding to the trigonal phase (around 168, 300, 524, and 582 cm^−1^) are well defined (see red arrows in Figure 2b). As observed, the strongest peak of the cubic phase disappears above 22.0 GPa, thus confirming that the A-type phase is the only one above that pressure. Interestingly, four additional broad bands (around 250, 400, 690, and 770 cm^−1^) are observed at 29.5 GPa. According to their frequencies, we tentatively attribute them to second-order Raman modes of the trigonal phase that could be the *A*_1*g*_^2^ − *A*_1*g*_^1^, *E_g_*^2^ − *E_g_*^1^, *E_g_*^1^ + *A*_1*g*_^2^, and *E_g_*^1^ + *E_g_*^2^ modes, respectively.

**Figure 3:**
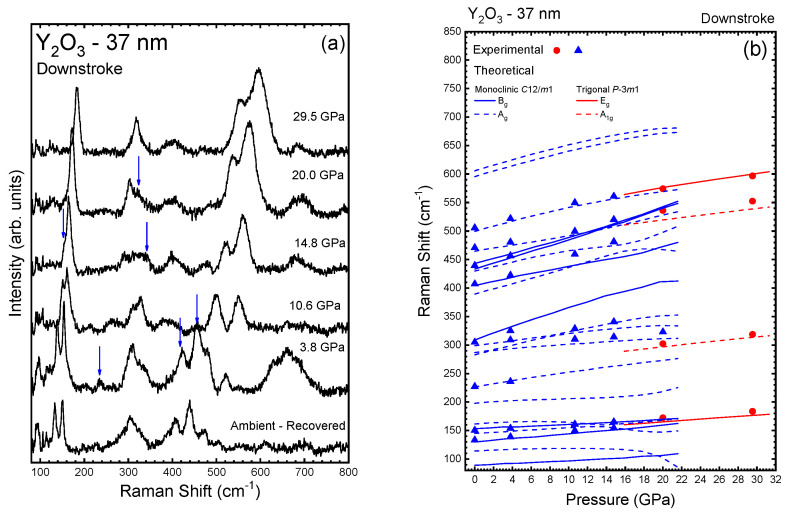
(**a**) Room-temperature Raman spectra of Y_2_O_3_-37 nm at selected pressures on downstroke. Blue arrows indicate the new peaks that appear on downstroke. (**b**) Pressure dependence of Raman-active modes of Y_2_O_3_-37 nm observed on downstroke.

Figure 3a shows the RS spectra of Y_2_O_3_-37 nm nanocrystals on downstroke. At 20.0 GPa and more notably at 14.8 GPa, new peaks can be detected (see blue arrows) apart from four peaks of the trigonal phase. The appearance of the new peaks is coincident with the disappearance of the mode of the trigonal phase near 300 cm^−1^ below 14.8 GPa. This result confirms the A-B PT in Y_2_O_3_-37 nm nanocrystals on downstroke. As in bulk material [19], the B-type phase is retained at ambient conditions in the Y_2_O_3_-37 nm nanocrystals as evidenced by the large number of Raman-active modes. The observation of a large number of Raman-active modes in recovered Y_2_O_3_-37 nm nanocrystals is in agreement with the RS spectrum of Y_2_O_3_ nanocrystals (~70 nm) recovered from 19 GPa studied by Dilawar et al. [47], where a broad band, corresponding to a partial amorphous sample, and a large number of Raman modes, likely corresponding to the monoclinic phase, were observed. However, we want to highlight that our explanation for the pressure behavior of Y_2_O_3_-37 nm nanocrystals, in which the B-type phase is recovered on downstroke, is different from that given by Dilawar et al., who considered that the Y_2_O_3_-70 nm nanocrystals recovered from 19 GPa consisted in a mixture of the cubic and trigonal phases [47].

Figure 2c and Figure 3b show the experimental pressure dependence of the Raman-active frequencies in the Y_2_O_3_-37 nm sample (symbols) compared to theoretical bulk Y_2_O_3_ (lines) on upstroke and downstroke, respectively. The good comparison between the experimental and theoretical results gives support to the suggested C-B-A PT sequence in Y_2_O_3_-37 nm nanocrystals on upstroke and has encouraged us to make a tentative assignment of the symmetry of each peak (Table 2, Table 3 and Table 4 for the C-, A-, and B-type phases, respectively). For comparison purposes, we also present in Table 2 the results of 70-nm C-type Y_2_O_3_ nanoparticles obtained by Sharma et al. [17].

In summary, our HP-RS measurements in Y_2_O_3_-37 nm nanocrystals do not exhibit a noticeable slope change above 8 GPa, unlike what was observed in nanotubes [25] and nanorods [48]. In the cubic phase, most Raman modes feature a non-linear pressure dependence as shown by theoretical calculations for bulk Y_2_O_3_. On the other hand, we conclude that our HP-RS measurements support the existence of a C-B PT in Y_2_O_3_-37 nm nanocrystals above 14.4 GPa on upstroke followed by a B-A PT above 20.6 GPa, with the C-type phase coexisting up to 22.0 GPa. On downstroke, an A-B PT occurs below 15 GPa. This PT sequence is different to those previously reported for many nanocrystals of similar size. For instance, Wang et al. [20] observed a C-A PT in 21-nm nanocrystals, while other authors have observed a PIA on upstroke even in nanocrystals of larger size than ours [21,47,53]. We must stress that our interpretation of the existence of the intermediate B-type phase prior to the appearance of the A-type phase in Y_2_O_3_-37 nm nanocrystals is supported by previous HP-RS results on Dy_2_O_3_-45 nm nanocrystals and Ho_2_O_3_-60 nm nanocrystals [54,55]. In this context, it is important to consider that both Dy, Ho, and Y have similar ionic radii, so similar PTs and PT pressures are expected in the two compounds [17]. More precisely, HP-RS results on Dy_2_O_3_-45 nm show the appearance of several bands at 14.6 GPa. Namely, a broad band around 530 cm^−1^ that develops into two bands at higher pressures, two bands around 200 cm^−1^ that develop into a single band at higher pressures, and especially a broad band around 310 cm^−1^ that disappears at 21.7 GPa. The existence of this broad band around 300 cm^−1^ at the low-energy tail of the main peak of the cubic phase is similar to that found in Y_2_O_3_-37 nm above 16.2 GPa and clearly can be attributed to the intermediate B-type phase, while the A-type phase in Dy_2_O_3_-40 nm nanocrystals and in Ho_2_O_3_-60 nm nanocrystals appears above 19 and 15.5 GPa, respectively [54,55]. Moreover, another broad band appears at 18.9 GPa in Y_2_O_3_-37 nm, where the main peak of the cubic phase was. This new broad band overlaps with the band near 300 cm^−1^ and locates as a shoulder of the two high-frequency peaks above 500 cm^−1^. This band also occurs in bulk Y_2_O_3_ above 17.9 GPa and also can be attributed to the intermediate B-type phase.

At this point we want to comment that the observation of the C-B-A PT in Y_2_O_3_ seems to be directly related to the particle size since the intermediate monoclinic phase has been observed in samples with more than 100 nm [42,56,57], while it has not been observed in nanocrystalline samples of smaller size [20,21,47,53]. In this context, it has been claimed that the cubic phase of nanocrystals exhibits an increased stability at HP with respect to the bulk material [20]. Therefore, since the C-B and B-A PTs in bulk Y_2_O_3_ are clearly observed around 13 and 20 GPa [19], there is no reason for not observing the C-B PT in nanocrystals provided that the increased stability of the cubic phase in nanocrystals does not exceed 10 GPa to higher pressures. In fact, previous papers claim that the PT in nanocrystals and nanotubes starts around 14-15 GPa [20,47], i.e., at pressures just slightly higher than those present in the bulk material [42].

**Figure 4:**
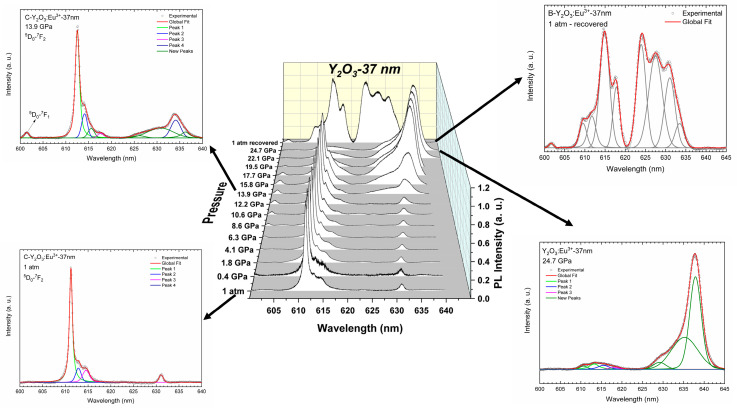
Room-temperature PL spectra of Y_2_O_3_:Eu^3+^-37 nm excited with 532 nm at selected pressures. The top spectra correspond to the recovered sample on downstroke. The inserts are more detailed representations of some spectra at specific pressures. The inserts also show the peaks obtained by deconvolution of the spectra using Voigt functions.

**Figure 5:**
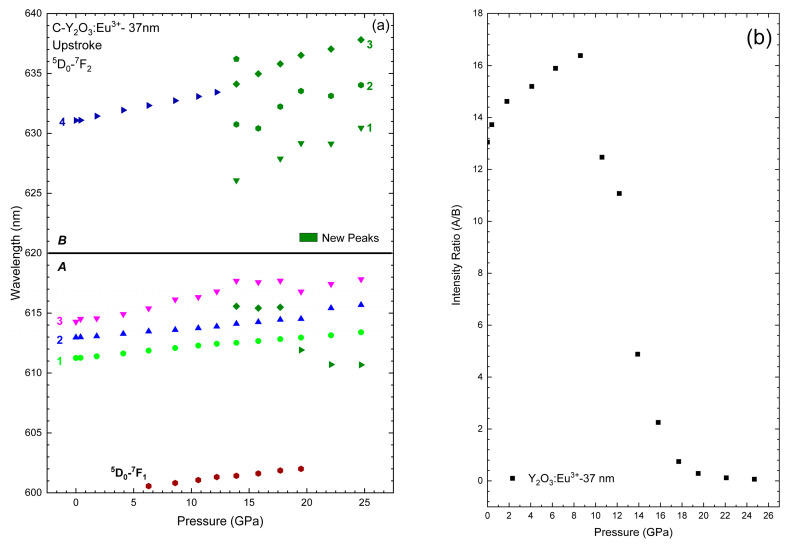
(**a**) Pressure dependence of the peaks related to the PL emissions originating from Y_2_O_3_:Eu^3+^-37 nm. Different colors and numbers are intended to differentiate between different PL bands present in the low-pressure phase (see Table 5). All new bands appearing at HP are plotted with dark green color and ordered with different numbers only for the high wavelength region. (**b**) Pressure dependence of the PL emission intensity ratio of the group A/B in Y_2_O_3_:Eu^3+^.

Finally, we performed a characterization of the PL properties Eu-doped Y_2_O_3_:Eu^3+^-37 nm nanocrystals under compression. Figure 4 shows the HP-PL measurements on Y_2_O_3_:Eu^3+^-37 nm nanocrystals in the region of the ^5^D_0_-^7^F_2_ electric dipole allowed transitions of Eu^3+^ (between 600 and 640 nm) [18,53,58,59] up to 24.7 GPa. Using Voigt functions, we could deconvolve the peaks as can be observed in the inserts of Figure 4. At ambient pressure (left-down insert of Figure 4), the PL spectra is dominated by a peak at 611.3 nm (Peak 1), that belongs to the C_2_ symmetry [60] and overlaps another two less intense at 612.9 nm (Peak 2) and 614.4 nm (Peak 3). Additionally, this phase is characterized by another peak at 631.1 nm (Peak 4). According to some published works [53,60,61], since the ^5^D_0_-^7^F_J_ (J = 2) splits into five (2J + 1) peaks, one would expect a fifth peak at ~627 nm that is not unambiguously observed at ambient conditions. As can be observed in Figure 4, all PL peaks shift to higher wavelength with pressure increase. The red shift of the PL peaks, more evident in Figure 5, can be related to the expansion of the *f* orbit of the Eu^3+^ ion that increases the covalence of the Eu-O bonds [58,60].

At 6.3 GPa, we can observe a peak at 600.6 nm that is attributed to the ^5^D_0_–^7^F_1_ transition of Eu^3+^. At 13.9 GPa, besides the initial four peaks, it is possible to detect four new peaks at 615.6 nm, 625.9 nm, 630.5 nm, and 636.2 nm (olive peaks on the top-left insert of Figure 4). As can be observed in Figure 5a, at this pressure, peak 4 also shows a significant change in its pressure coefficient, which may indicate the beginning of the PT. Previous studies [53,60] have suggested that the A-type structure has three main peaks in the region of 620–640 nm. Those studies could indicate that the peaks at 625.9 nm, 630.5 nm, and 633.9 nm belong to this phase. However, the B-type phase also shows bands in that region (see top-right insert of Figure 4). Therefore, those peaks could also correspond to the B-type phase. As previously stated, PL measurements are more sensitive to structural variations than RS and XRD, which justifies the fact that we could observe the B-type phase at lower pressures than in previous measurements. In fact, two new peaks appear above 20 GPa, thus suggesting the PT to the A-type phase, while other peaks disappear (olive symbols in Figure 5a). This result clearly indicates that the new peaks appearing above 13.9 GPa most likely correspond to the intermediate B-type phase, while the PT to the A-type phase is observed above 20 GPa.

At this point, our results can be compared to those of Zhang et al. [60] who studied bulk Y_2_O_3_:Eu^3+^ at HP and subdivided this PL region into two zones: the low-energy zone between 600 and 620 nm (A-zone) and the high-energy zone between 620 and 640 nm (B-zone). Our PL spectra at low pressures are similar to those reported by Zhang et al. for bulk Y_2_O_3_:Eu^3+^ and clearly correspond to the cubic phase [60]. Increasing pressure, we can see an increase in the intensity of the peaks in the B-zone concomitant with the decrease in the peaks in the A-zone. In fact, our PL spectrum at 24.7 GPa is similar to those reported for the trigonal phase in bulk Y_2_O_3_:Eu^3+^ above 23.5 GPa [60], which consists of three broad bands in the B-zone (centered on 629.1 nm, 635.1 nm, and 637.8 nm) and three residual bands related to the C-type structure in the A-zone (right-down insert of Figure 4). Here, we can comment that, as can be observed in the right-down insert of Figure 4, we cannot fit the residual band in the A-zone without the peak at ~610 nm. Actually, above 19.5 GPa, we need to add this peak in order to obtain a reasonable fit. As can be observed in Figure 5a, this peak presents a negative pressure coefficient, a common behavior in many bands at pressures close to a PT, that reinforces our conclusion regarding the PT to the A-type phase occurring near 20 GPa.

Returning to ambient pressure, the spectrum presents five peaks in the A-zone (centered on 609.6 nm, 611.7 nm, 614.9 nm, and 617.8 nm) and four in the B-zone (centered on 623.9 nm, 627.5 nm, 631.0 nm, and 633.5 nm—right-top insert of Figure 4). The recovered spectrum is similar to that presented by other works, and is related to Eu^3+^ transitions in B-type Y_2_O_3_ [53,60]. In conclusion, based on all the results presented so far, we attribute the changes in the PL spectra of Y_2_O_3_:Eu^3+^-37 nm to the C-B-A PT sequence on upstroke, and the A-B transition on downstroke in good agreement with HP-XRD and HP-RS measurements in Y_2_O_3_-37 nm.

The pressure coefficients of the fitted peaks are presented in Table 5 and are in good agreement with the values presented by Bai et al. and Zhang et al. [53,60]. These authors attributed the pressure-induced changes in the relative intensities of the peaks in the A- and B-zones (A/B ratio) in bulk Y_2_O_3_ to the breaks in the crystalline field due to the PTs. In our 37 nm sample, the intensity of the peaks of the A-zone tends to increase when compared to that of the peaks of the B-zone up to 6.3 GPa (see Figure 5b). From ~6.3 GPa to ~19 GPa this trend is reversed, i.e., the intensity of the peaks of the B-zone show a relatively high rate of growth in relation to the peaks of the A-zone. Finally, above ~19 GPa, the peaks of the B-zone dominate the spectrum, and the A/B ratio tends to zero. This behavior can be directly associated to the C-B-A PT sequence on upstroke. Therefore, we can conclude that the decrease in the A/B ratio above 8 GPa is related to the progressive transition of the C-type structure to the B-type structure observed above 14 GPa in our PL measurements. In summary, our HP-PL measurements on Y_2_O_3_:Eu^3+^-37 nm confirm the same PT sequence as in pure Y_2_O_3_-37 nm and in bulk Y_2_O_3_. Therefore, our results suggest that dopants up to 1 at. wt% barely affect the stability of the Y_2_O_3_ nanocrystals at HP.

### 3.2. Y_2_O_3_-6 nm and Y_2_O_3_:Eu^3+^-6 nm Nanocrystals

**Figure 6:**
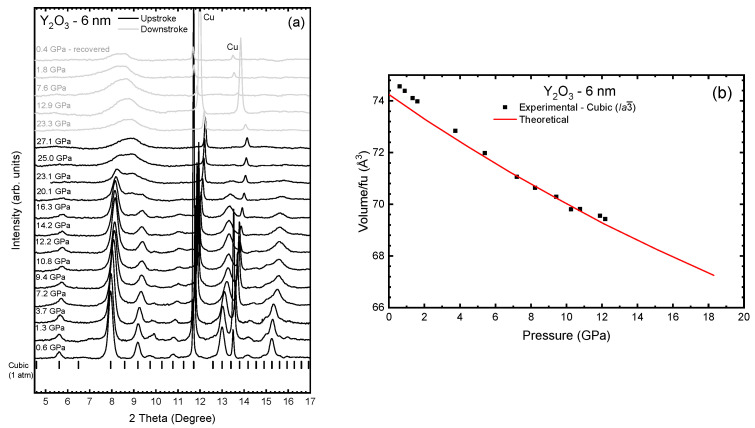
(**a**) Room-temperature XRD measurements of Y_2_O_3_-6 nm at selected pressures on upstroke (black lines) and downstroke (grey lines). Black symbols in figure (**a**) indicate the position of the diffraction peaks of the C-type phase according to the data obtained from our theoretical calculation. (**b**) Experimental (black symbols) and theoretical (red line) unit-cell volume pressure dependence of the cubic phase of Y_2_O_3_-6 nm during upstroke.

Figure 6a presents the HP-XRD measurements up to 27.1 GPa of the Y_2_O_3_-6 nm nanoparticles. All diffraction peaks followed at HP correspond to the cubic phase, thus reinforcing the absence of unwanted contaminants. The diffraction peaks shift to higher angles at HP, thus indicating a decrease in the lattice parameters at HP and showing a significant broadening concomitant with an intensity decrease at HP. Due to this behavior, we could perform Le Bail refinements with relative confidence up to 12.2 GPa. Above this pressure, the peaks lose definition and, at pressures higher than 23.1 GPa, we can only detect broad bands, thus indicating the PIA of the samples. This is rather different behavior under compression than Y_2_O_3_-37 nm nanoparticles. On releasing pressure, no defined peaks could be observed in the diffractogram, thus confirming that the PIA process is irreversible in Y_2_O_3_-6 nm nanoparticles (Figure 6a). A size-dependent PIA has already been observed in a number of Y_2_O_3_ and Y_2_O_3_:Eu^3+^ nanoparticles, nanotubes, and nanorods [20,21,22,23,24,25,47,62,63].

On the other hand, our interpretation of the existence of the B-type and A-type phases in Y_2_O_3_-6 nm samples prior to PIA is supported by the observation of the B-type phase prior to PIA in Ho_2_O_3_-14 nm nanocrystals under compression [64]. It must be recalled that Ho has a similar ionic radius to Y, so similar PTs and PT pressures are expected in both compounds. In Ho_2_O_3_-14 nm nanocrystals, the B-type phase was found above 14.7 GPa followed by a PIA above 21.4 GPa. Additionally, the PIA of Y_2_O_3_ nanoparticles and other RE oxides has already been observed [17,47]. This suggests that *f*-electrons could play a role in the bonding and formation of the HP trigonal phase, as has already been suggested [65].

It must be stated that no clear signal of B- or A-type Y_2_O_3_ could be observed in the HP-XRD measurements of Y_2_O_3_-6 nm. However, HP-XRD measurements on Y_2_O_3_-6 nm nanoparticles showed the first sign of PIA at 16.3 GPa with the appearance of a peak at 8.9°, which later evolved into a broad band. Interestingly, the first indications of B-type Y_2_O_3_ were detected at a similar pressure using HP-RS measurements (see discussion below). The simultaneous appearance of PIA in Y_2_O_3_-6 nm and the C-B PT in bulk could logically align with the concept that PIA is a phenomenon occurring due to the hindrance of a crystalline-to-crystalline PT, typically at relatively low temperatures, such as room temperature [63,66]. It is difficult to identify if the recovered amorphous phase is similar to the C-type or the B-type phase since in both phases the strongest diffraction peak is located near 8° at room pressure [19]. However, the broad band around 8° (coincident with the three diffraction peaks of the monoclinic phase [19]) and the absence of bands near 5.6, 9.2, 13, and 15.3° (characteristic of the cubic phase [19]) suggest that the amorphous phase recovered at 0.4 GPa in Figure 6a could be attributed to an amorphous sample which shows ordering similar to the local coordination typical of the B-type phase, or to an amorphous sample showing local ordering similar to that of the B- and C-type phases but with predominance of the B-type phase.

The experimental zero-pressure volume, V_0_, bulk modulus, B_0_, and its pressure derivative, B_0_′, were obtained from a 2^nd^-order BM-EoS to fit our P-V data (see Figure 6b) in the quasi-hydrostatic pressure range of the PTM (0–10 GPa). The experimental results for the Y_2_O_3_-6 nm sample were added in Table 1 for comparison with bulk and Y_2_O_3_-37 nm samples. For comparison purposes, once again we used the unit-cell volume per formula unit. The fit of the experimental data yields V_0_/Z = 74.88(7) Å^3^ and B_0_ = 127(2) GPa. The axial compressibility (κ_a_ = 3.3(2) × 10^−3^ GPa^−1^) was obtained from a modified BM-EoS fit [67] of the experimental data in the quasi-hydrostatic pressure range of the PTM (0–10 GPa) and is also reported in Table 1 for comparison purposes. Compared to bulk [19] and 37-nm nanoparticles, we observed a higher axial compressibility for the 6-nm nanoparticles, which justifies its smaller B_0_ with respect to the samples with larger size grains. In addition, we found that 6-nm nanoparticles also have higher V_0_ values (Table 1), which is in line with the B_0_ behavior. There are several studies showing the dependence of B_0_ on particle size. Some of them claim that the smaller the nanoparticle size is, the higher the B_0_ will be [68,69,70]. Others authors suggest that B_0_ must decrease with the particle size [71,72]. Therefore, there is still no consensus on how B_0_ is influenced by the particle size, new studies with different types of nanoparticles being necessary to better understand which are the main mechanisms responsible for a higher or lesser compressibility depending on the particle size. In any case, it seems clear that there is an inverse relation between V_0_ and B_0_. Therefore, nanocrystalline samples with different V_0_ than bulk must show different B_0_.

**Figure 7:**
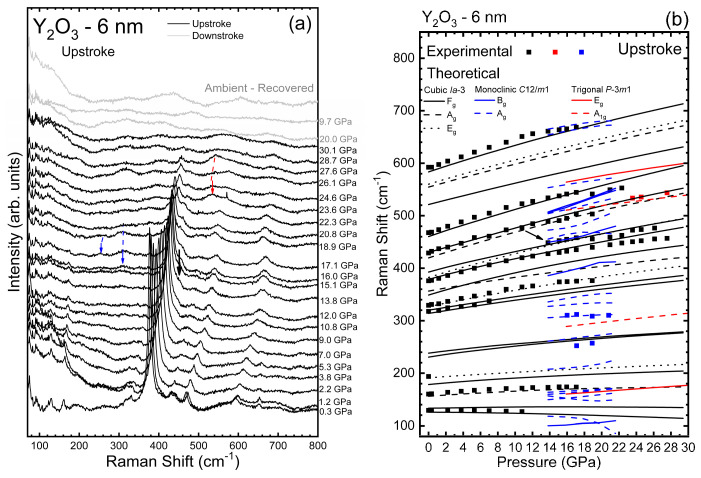
(**a**) Room-temperature Raman spectra of Y_2_O_3_-6 nm at selected pressures on upstroke (black) and downstroke (grey). The black and red spectra are related to the upstroke and downstroke measurements, respectively. Black, blue, and red arrows indicate new peaks not observable below 13.8 GPa that can be related to the C-, B-, and A-type phases of the Y_2_O_3_. (**b**) Pressure dependence of the experimental (symbols) Raman-active frequencies of Y_2_O_3_-6 nm. Theoretical Raman-active frequencies of A-, B-, and C-type bulk Y_2_O_3_ (red, blue, and black lines, respectively) are also plotted for comparison.

In order to complement the structural characterization, Figure 7a presents the HP-RS spectra of Y_2_O_3_-6 nm at selected pressures up to 30.1 GPa. Similar to the Y_2_O_3_-37 nm and Y_2_O_3_-Bulk samples [19], all peaks, except the peak initially at 129.3 cm^−1^, tend to shift to higher frequencies at HP. Above ~7 GPa, the less intense peaks start to disappear, while the more intense peaks lose intensity and broaden. At 15.1 GPa, four peaks from the initial phase have already disappeared and it is possible to observe a new peak at ~445 cm^−1^ (black arrow in Figure 7). By comparing with the theoretical calculations, we believe that this peak is related to an *F_g_*^10^ mode of C-type Y_2_O_3_ that initially should be at 392.7 cm^−1^ and was overlaid by the *F_g_*^9^ mode initially at 378.8 cm^−1^. At 16.0 GPa and 17.1 GPa, two broad bands can be observed around 310.8 cm^−1^ and 252 cm^−1^, respectively (blue arrows in Figure 7a). Both peaks disappear at 20.8 GPa and, according to our theoretical calculation, can be related to the *A_g_* vibrational modes of B-type Y_2_O_3_ (see Figure 7b). This result contrasts with the current view of pressure-induced PTs in Y_2_O_3_ nanocrystals because the presence of B-type Y_2_O_3_ Raman-active modes during upstroke was not previously observed in nanoparticles smaller than 16 nm, to our knowledge.

At 23.6 GPa, we could detect the formation of a broad band at 532.3 cm^−1^ that disappears at 27.6 GPa (red arrow in Figure 7a). According to our theoretical calculations, this band can be related to the *A*_1*g*_ mode of A-type Y_2_O_3_ (Figure 7b). Finally, above 28.7 GPa, we could not detect any peak. According to the XRD results previously presented, this behavior can be related to the complete PIA of the sample, which also is not reversible since the recovered sample does not present any Raman peak (see grey spectra of Figure 7a). In summary, we conclude that there is an irreversible PIA of Y_2_O_3_ nanoparticles with sizes around 6 nm.

In this context, we must comment that our interpretation of the existence of the B-type and A-type phases in Y_2_O_3_-6 nm samples that appear prior to complete PIA according to HP-RS measurements is supported by the previous observation of the B-type phase prior to PIA in Ho_2_O_3_-14 nm nanocrystals under compression [64]. Note also that Ho has a similar ionic radius to Dy and Y, so similar PTs and PT pressures are expected in these Ln_2_O_3_ compounds. In Ho_2_O_3_-14 nm nanocrystals, the B-type phase was found above 14.7 GPa followed by a PIA above 21.4 GPa. Additionally, the PIA of Y_2_O_3_ nanoparticles and other RE-SOs has already been observed [17,47]. This result suggests that *f*-electrons could play a role in the bonding and formation of the HP trigonal phase in RE-SOs, as has been already suggested [65].

It must be also stressed that PIA in the smallest nanoparticles is a common phenomenon since it has been argued that decreasing the nanocrystal size leads to considerable strains and structural distortions that result even in a mixture of crystalline and amorphous phases at ambient conditions [63,66]. Therefore, it is not surprising that full amorphization occurs at HP in the smallest nanoparticles since pressure can further promote the distortion of the lattice, especially at the boundary of a PT. Unfortunately, there are not enough HP studies on nanoparticles of RE-SOs with sizes of the order of 10 nm or less to unambiguously certify PIA in all these compounds (note that previous HP studies have been mostly performed with nanoparticles ranging from 40 to 90 nm).

Figure 7b shows the pressure dependence of the experimental and theoretical Raman-active frequencies in Y_2_O_3_-6 nm. Up to ~16 GPa, there is a good agreement between the experimental and theoretical results, which encouraged us to propose that the observed modes correspond to the cubic phase (Table 2). However, above this pressure, the positions of the peaks that can still be detected present anomalous behaviors when compared to those expected by theoretical calculations (Figure 7b). We think that this behavior may be related to the loss of symmetry induced by the beginning of the PIA process. Comparing the results of Table 2 with the results presented in ref. [19], it can be observed that most phonon frequencies of the cubic phase in bulk and nanocrystalline Y_2_O_3_ decrease as the particle size decreases. This result agrees with the results of Beck et al. [73], who attributed the decrease in all Raman-active frequencies of Y_2_O_3_ to the increase in the Y-O bond length upon decreasing the nanocrystal size due to the increase in the lattice parameter of the cubic phase in nanocrystals as compared to the bulk material [73,74].

**Figure 8:**
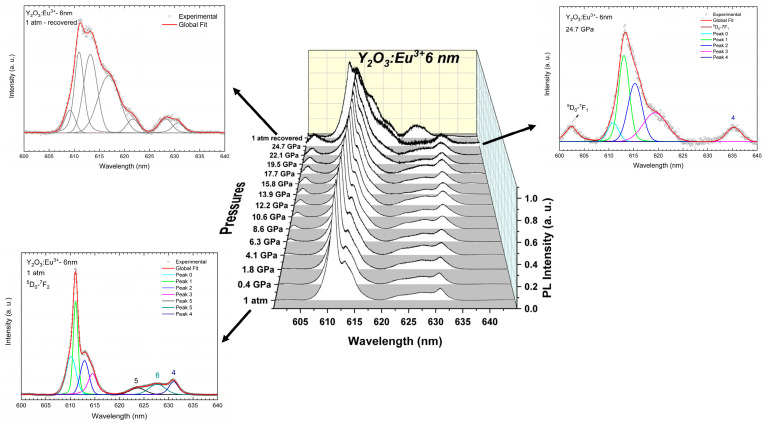
Room-temperature normalized PL spectra of Y_2_O_3_:Eu^3+^-6 nm excited with 532 nm at selected pressures. The top spectra correspond to the recovered sample. The inserts are more detailed representations of some spectra at specific pressures. The inserts also show the peaks obtained by deconvolution of the spectra using Voigt functions.

**Figure 9:**
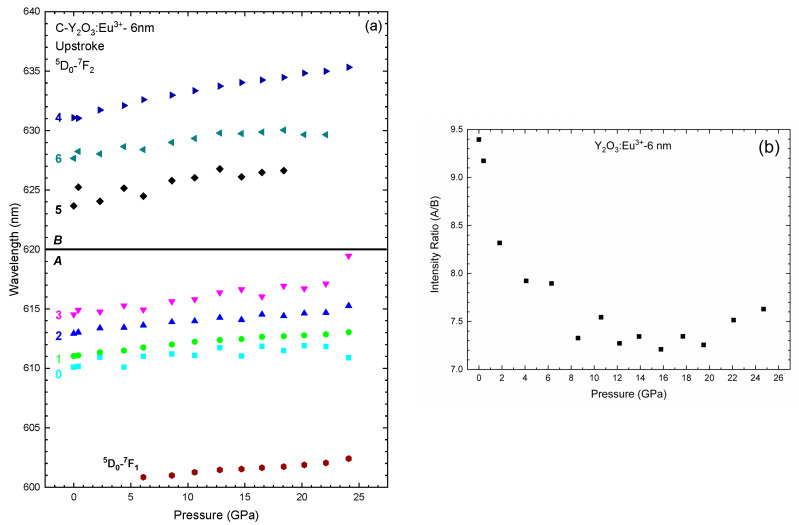
(**a**) Pressure dependence of the wavelengths related to the PL emissions in Y_2_O_3_:Eu^3+^-6 nm. (**b**) Pressure dependence of the PL emission intensity ratio of the group A/B in Y_2_O_3_:Eu^3+^.

Figure 8 and Figure 9a present the PL spectra from 600 to 640 nm (^5^D_0_-^7^F_2_ transition) and the pressure evolution of the wavelengths for the different peaks of Y_2_O_3_:Eu^3+^-6 nm, respectively. As for Y_2_O_3_:Eu^3+^-37 nm, we have modelled the PL peaks of Y_2_O_3_:Eu^3+^-6 nm using Voigt functions. As can be observed in the left-down insert of Figure 8, the PL spectrum at ambient pressure is composed of seven peaks. This includes the four peaks also observed in the Y_2_O_3_:Eu^3+^-37 nm sample; i.e., those centered at 611.0 nm (Peak 1), 612.9 nm (Peak 2), 614.5 nm (Peak 3), and 631.1 nm (Peak 4), plus three new peaks centered at 610.1 nm (Peak 0), 623.6 nm (Peak 5), and 627.7 nm (Peak 6). As already commented, the peak at 627.7 nm is one of the expected peaks related to the ^5^D_0_–^7^F_2_ transition of the Eu^3+^ ion [53,60]; however, the nature of the peaks at 610.1 nm and 623.6 nm is still unclear, requiring further investigation of the PL properties of Eu^3+^ when incorporated into nanoparticles of different sizes (an issue that is beyond the scope of the present work).

As pressure increases, the PL peaks tend to shift to higher wavelengths at practically the same rate as the Y_2_O_3_-37 nm sample (see Table 5). Notably, most of the PL peaks of the cubic phase are still observed up to 24.7 GPa but with smaller intensity than in the Y_2_O_3_-37 nm sample (right insert of Figure 8). From the initial peaks, only peaks 5 and 6 disappear at 19.5 GPa and 22.1 GPa, respectively. On downstroke from 24.7 GPa, the PL spectrum at 1 atm shows seven peaks (left-up insert of Figure 8) as the original cubic phase; however, one can observe significant differences between both PL spectra that indicate that the sample does not return to the original cubic phase. This result agrees with the irreversible PIA process observed in our HP-RS and HP-XRD measurements. It must be noted that the PL spectrum at 1 atm in the recovered sample shows two main peaks near 611 and 615 nm, which are coincident with the most intense PL peaks of the C-type and the B-type phases, respectively (see Figure 8). Therefore, HP-PL measurements in Y_2_O_3_-6 nm confirm the PIA process and that the recovered sample is likely an amorphous sample which shows some local ordering similar to the local coordination typical of B- and C-type phases, in agreement with the results obtained from the HP-XRD measurements previously commented.

As can be seen in Figure 9a, the pressure dependence of the PL peaks of Y_2_O_3_-6 nm does not show significant variations in the pressure coefficients. This result is similar to that observed in the 37 nm sample (Figure 5a). Thus, we suggest that the PL spectrum observed at 24.7 GPa corresponds to an amorphized cubic phase. In order to reinforce this statement, we carried out the same analysis of the ratio between the intensity of the peaks in A and B regions. As can be seen in Figure 9b, the A/B ratio tends to decrease to ~8 GPa and remains practically constant above that pressure. This result is completely different to that obtained in Y_2_O_3_-37 nm, where the ratio tends to 0 at HP. Therefore, the stabilization of the A/B ratio above 8 GPa seems to evidence the PIA process followed by C-type Y_2_O_3_-6 nm; i.e., it is completely different behavior under compression with respect to Y_2_O_3_-37 nm that undergoes the C-B-A PT sequence. Moreover, if we assume that the change in the A/B ratio is due to the C- to B-type PT and that the ratio tends to zero when the PT is completed, the constant value (around 7) of the A/B ratio in 6-nm nanocrystals indicates that the C-B PT is far from completed irrespective of the pressure applied. Finally, on decreasing pressure the PL spectrum of Y_2_O_3_-6 nm shows bands that are between those of C- and B-type Y_2_O_3_ (see Figure 8). Therefore, our HP-PL study confirms that the recovered sample is likely an amorphous sample which shows some local ordering similar to the local coordination typical of the B- and C-type phases. In this regard, we must comment that the local ordering of the amorphous sample means that Y atoms must be in an almost sixfold coordination as most Y atoms are in the B- and C-type phases. Note that the A-type phase shows all Y atoms with sevenfold coordination. This different average cation coordination allows us to explain the similitudes between the XRD pattern and the RS and PL spectra of the amorphous phase and those of the B- and C-type phases.

## 4. Conclusions

We have reported a joint HP experimental and theoretical study of the structural, vibrational, and photoluminescence properties of pure and Eu^3+^-doped C-type Y_2_O_3_ nanoparticles with two very different sizes (6 nm and 37 nm). HP-XRD, HP-RS, and HP-PL measurements in pure and Eu^3+^-doped Y_2_O_3_ nanoparticles have indicated that the behavior of nanoparticles under compression depends on the average particle size. This multi-technique approach on the same samples with different average size distributions is the main strength of this work. Nanoparticles with an average particle size of ~37 nm show the same pressure-induced PT sequence on upstroke and downstroke as the bulk sample [19], unlike what has been found in many other previous works. We find it difficult to explain why our results differ from previous ones, but it is clear that using the same experimental conditions in a multi-technique approach is a prerequisite for a consistent understanding of the behavior of nanoparticles under compression. On the other hand, nanoparticles with an average particle size of ~6 nm undergo an irreversible PIA (starting above 16 GPa and ending above 24 GPa). On downstroke from 25 GPa, 6-nm nanocrystals show an amorphous phase that exhibits local ordering akin to the local sixfold coordination typical of the B- and C-type phases (perhaps with predominance of the B-type phase). Consequently, our findings for 6 nm nanocrystals show a different behavior than bulk and 37 nm nanocrystals; i.e., the smallest nanoparticles show a PIA that is not observed in the largest ones. This size-dependent response of Y_2_O_3_ nanocrystals aligns with the analogous behaviors observed in bulk and larger nanocrystals under compression, as well as the previously noted size-dependent behavior of nanocrystals under compression.

Finally, as regards the vibrational and photoluminescent properties of yttria nanocrystals at HP, we have not observed any noticeable change in the slope of the Raman frequencies above 8 GPa, unlike in previous works, but we have observed a drastic change in the PL intensity ratios above 8 GPa. We have concluded that the decrease in the PL intensity ratio above 8 GPa in nanocrystals is not simply related to the distortion of the YO_6_ octahedra in C-type Y_2_O_3_ as previously assumed. Instead, we attribute it to the decrease in C-phase and an increase in B-phase clearly observed above 14 GPa in our PL measurements. Moreover, the HP behavior of the intensity ratio of the PL peaks of two zones (A and B) of the C-type phase has been found to be different in the nanoparticles of 6 and 37 nm due to the different structural behavior of these nanoparticles at HP. In summary, this work provides a consistent understanding of the structural, vibrational, and photoluminescent properties of Y_2_O_3_ nanoparticles under compression with different average particle sizes that can help in understanding the effect of pressure on the properties of other Ln_2_O_3_ nanoparticles, in particular those whose cation has a similar ionic radius as Y, such as Tb, Dy, Ho, and Er. The different behavior under compression shown by the nanoparticles of 6 and 37 nm clearly point to the important influence of particle size on the structural evolution of Y_2_O_3_ under pressure. Notably, we have provided measurements under compression in Y_2_O_3_ nanoparticles with an average size below 10 nm which can be a guide for future high pressure studies in nanoparticles of RE-SOs of similar size, which have been barely addressed.

## Figures and Tables

**Table 1:** EOS parameters and axial compressibility ($\kappa_{a}=\frac{-1}{a}\frac{\partial a}{\partial P}$) at ambient pressure of experimental Y_2_O_3_-37 nm and Y_2_O_3_-6 nm samples, as well as of theoretical cubic, trigonal, and monoclinic Y_2_O_3_. The variation $\frac{\partial a}{\partial P}$ was obtained using the Murnaghan equation of state Δa0a0=1+B0′PB0−13B0′−1, where $B_{0}$ and $B_{0}^{\prime }$ are the bulk modulus and its pressure derivative of the *a*-axis at atmospheric pressure. In order to simplify the comparison between the structures, V_0_ is presented per formula unit (Z): cubic (Z = 16); trigonal (Z = 1); monoclinic (Z = 6).

	V_0_/Z (Å^3^)	B_0_ (GPa)	$B_{0}^{\prime }$	κ_a_ (10^−3^ GPa^−1^)
Experimental				
Y_2_O_3_—Bulk ^a^ (Up to 12 GPa)	74.517 (8)	147 (1)	3.2 (3)	2.3 (5)
Y_2_O_3_-37 nm (Up to 12 GPa)	74.82 (5)	144 (7)	3 (1)	2.5 (5)
Y_2_O_3_—6 nm (Up to 10 GPa)	74.88 (7)	127 (2)	4 (Fixed)	3.3 (2)
Theoretical				
C-type Bulk Y_2_O_3_ (Up to 12 GPa) ^a^	74.24 (1)	154 (1)	3.5 (2)	2.18 (8)

^a^ Ref. [19].

**Table 2:** Experimental (Y_2_O_3_-37 nm and Y_2_O_3_-6 nm) and DFT-PBEsol theoretical zero-pressure wavenumbers (in cm^−1^) and linear pressure coefficients (in cm^−1^/GPa) of Raman-active modes of the C-type Y_2_O_3_. The experimental values of Sharma et al. [17] have been also added for comparison.

*Symmetry*	Experimental				Theoretical		Sharma et al.	
	37 nm		6 nm					
	*ω*_0_	*dω*/*dP*	*ω*_0_	*dω*/*dP*	*ω*_0_	*dω*/*dP*	*ω*_0_	*dω*/*dP*
*F_g_* ^1^	129.7	−0.27	129.3	−0.19	125.7	−0.34		
*F_g_* ^2^					133.4	0.11		
*A_g_* ^1^	161.8	0.93	160.2	0.80	156.1	0.74		
*F_g_* ^3^					178.5	0.97		
*E_g_* ^1^	194.4	1.26	193.7	-	191.2	0.92		
*F_g_* ^4^					230.6	1.73		
*F_g_* ^5^					238.2	1.50		
*F_g_* ^6^	316.9	2.44	317.8	2.74	313.9	2.29	316	
*F_g_* ^7^					320.1	2.37		
*E_g_* ^2^	329.8	3.06	329.3	3.18	326.8	2.81	331	
*F_g_* ^8^					348.9	3.57		
*A_g_* ^2^					356.4	2.31		
*F_g_* ^9^	377.2	3.65	376.1	2.87	378.8	3.51	381.3	3.79
*E_g_* ^3^					382.4	4.10		
*F_g_* ^10^	408.0 *	3.25			392.7	3.58		
*A_g_* ^3^					419.7	4.47		
*F_g_* ^11^	430.4	4.44	429.1	4.01	430.1	4.42		
*F_g_* ^12^	468.9	4.95	467.7	3.98	460.3	4.80	472.0	5.02
*F_g_* ^13^					521.4	3.86		
*A_g_* ^4^					554.6	4.11		
*E_g_* ^4^					560.0	4.30		
*F_g_* ^14^	592.4	4.78	592.4	3.71	583.4	4.63	598.1	4.58

* This peak was observed from 6.4 GPa.

**Table 3:** Experimental (Y_2_O_3_-37 nm) and DFT-PBEsol theoretical wavenumbers (in cm^−1^) and linear pressure coefficients (in cm^−1^/GPa) for the Raman-active modes of A-type Y_2_O_3_. Fits were performed from the pressure where the peaks are observed and the value of the frequency ω_22GPa_ corresponds to the pressure of 22 GPa.

*Symmetry*	Experimental		Theoretical	
	*ω*_22*GPa*_	*dω*/*dP*	*ω*_22*GPa*_	*dω*/*dP*
*E_g_* ^1^	184.7	1.35	168.4	1.16
*A*_1*g*_^1^	319.4	2.10	300.5	1.71
*A*_1*g*_^2^	553.1	2.27	523.7	2.00
*E_g_* ^2^	596.7	2.27	581.6	2.42

**Table 4:** Experimental (Y_2_O_3_-37 nm) and DFT-PBEsol theoretical zero-pressure wavenumbers (in cm^−1^) and linear pressure coefficients (in cm^−1^/GPa) for the Raman-active modes of B-type Y_2_O_3_.

*Symmetry*	Experimental		Theoretical	
	*ω*_0_	*dω*/*dP*	*ω*_0_	*dω*/*dP*
*B* *_g_* ^1^			89.2	0.77
*A* *_g_* ^1^			114.2	0.37
*B* *_g_* ^2^	133.3	1.45	130.0	1.40
*A* *_g_* ^2^			145.0	1.00
*B* *_g_* ^3^	150.3	1.01	153.2	0.72
*A* *_g_* ^3^			161.5	0.08
*A* *_g_* ^4^			197.7	0.71
*A* *_g_* ^5^			226.3	2.54
*A* *_g_* ^6^			282.8	1.58
*A* *_g_* ^7^			286.7	3.33
*A* *_g_* ^8^			298.8	2.53
*B* *_g_* ^4^			308.7	5.58
*A* *_g_* ^9^			389.5	4.69
*B* *_g_* ^5^	407.0	3.99	404.3	3.30
*A* *_g_* ^10^			429.7	3.39
*B* *_g_* ^6^			433.3	5.12
*B* *_g_* ^7^	439.5	2.40	443.0	4.60
*A* *_g_* ^11^	470.7	3.24	464.6	2.73
*A* *_g_* ^12^	505.6	3.79	499.4	4.06
*A* *_g_* ^13^			595.5	4.36
*A* *_g_* ^14^			605.7	4.44

**Table 5:** Wavelengths and their pressure coefficients for the ^5^D_0_–^7^F_2_ PL emission peaks of Eu^3+^ observed in C-type Y_2_O_3_:Eu^3+^-37 nm and Y_2_O_3_:Eu^3+^-6 nm samples. For comparison purposes, the data obtained by Zhang et al. [60] and Bai et al. [53] are also presented.

Peak	Y_2_O_3_:Eu^3+^-37 nm		Y_2_O_3_:Eu^3+^-6 nm		Zhang et al.		Bai et al.		
					Bulk		λ_0_ (nm)	Bulk	20 nm Nanoparticle
	λ_0_ (nm)	dλ/dP (nm/GPa)	λ_0_ (nm)	dλ/dP (nm/GPa)	λ_0_ (nm)	dλ/dP (nm/GPa)		dλ/dP (nm/GPa)	dλ/dP (nm/GPa)
^5^D_0_–^7^F_1_	600.6	0.11 (1)	600.8	0.08 (1)					
0	-	-	610.1	0.06 (1)					
1	611.2	0.09 (1)	611.0	0.08 (1)	611.9	0.098	611.4	0.101	0.102
2	612.9	0.10 (1)	612.9	0.08 (1)					
3	614.3	0.15 (2)	614.5	0.14 (2)					
4	631.1^a^	0.20 (1) ^a^	631.1	0.17 (1)			631.4	0.198	0.184
5	-	-	623.6	0.14 (2)					
6	-	-	627.7	0.10 (1)					

^a^ up to 13.9 GPa.

## Data Availability

Data are available upon request to interested researchers.

## Supplementary Materials

The following supporting information can be downloaded at: https://www.mdpi.com/article/10.3390/nano14080721/s1, Figure S1. RS measurements of bulk Y_2_O_3_ and pure and Eu^3+^-doped Y_2_O_3_ nanocrystals at ambient pressure (outside the DAC). Peaks marked with * are related to the luminescence of Eu^3+^ ions in the Y_2_O_3_ lattice. Figure S2. (a) Room-temperature Raman spectra of Y_2_O_3_:Eu^3+^-37 nm at selected pressures on upstroke. The upper spectrum is related to the recovered sample after decompression and the * indicates a peak unrelated to the initial phase. (b) Pressure dependence of the experimental (symbols) Raman-active frequencies of Y_2_O_3_:Eu^3+^-37 nm on upstroke. Blue lines represent the theoretical Raman-active frequencies of bulk C-type Y_2_O_3_. Red symbols represent peaks related to the Eu^3+^ luminescence. Figure S3. (a) Room-temperature Raman spectra of Y_2_O_3_:Eu^3+^-6 nm at selected pressures on upstroke. The upper spectrum is related to the recovered sample after decompression. (b) Pressure dependence of the experimental (symbols) Raman-active frequencies of Y_2_O_3_:Eu^3+^-6nm on upstroke. Blue lines represent the theoretical Raman-active frequencies of bulk C-type Y_2_O_3_. Red symbols represent peaks related to the Eu^3+^ luminescence.
