# Supplementary material for: Size-Dependent High-Pressure Behavior of Pure and Eu3+-Doped Y2O3 Nanoparticles: Insights from Experimental and Theoretical Investigations
Source: Nanomaterials (Basel). 2024 Apr 20;14(8):721. doi: 10.3390/nano14080721 (PMC11054519; doi:10.3390/nano14080721)
Supplement: Supplementary file 1 [file nanomaterials-14-00721-s001.zip › nanomaterials-2965155-supplementary.pdf]

nanomaterials

MDPI

Supplementary Material of

Size-Dependent High-Pressure Behavior of Pure and Eu^{3+}-Doped Y_2O_3 Nanoparticles: Insights from Experimental and Theoretical Investigations

André Luis de Jesus Pereira [1,2,*], Juan Ángel Sans [1], Óscar Gomis [3], David Santamaría-Pérez [4], Sudeshna Ray [5], Armstrong Godoy, Jr. [2], Argemiro Soares da Silva-Sobrinho [2], Plácida Rodríguez-Hernández [6], Alfonso Muñoz [6], Catalin Popescu [7] and Francisco Javier Manjón [1,*]

[1] Instituto de Diseño para la Fabricación y Producción Automatizada, MALTA Consolider Team, Universitat Politècnica de València, 46022 València, Spain; juasant2@upvnet.upv.es
[2] Laboratório de Plasmas e Processos—LPP, Instituto Tecnológico de Aeronáutica—ITA, São José dos Campos 12228-900, Brazil; godoyajr@gmail.com (A.G.J.); argemirosss@gmail.com (A.S.d.S.-S.)
[3] Centro de Tecnologías Físicas, MALTA Consolider Team, Universitat Politècnica de València, 46022 València, Spain; osgohi@fis.upv.es
[4] Departament de Física Aplicada-ICMUV, MALTA Consolider Team, Universitat de Valencia, 46100 Burjassot, Spain; david.santamaria@uv.es
[5] Department of Chemistry, Rabindranath Tagore University, Bhopal 464993, Madhya Pradesh, India; sudeshnaskype@gmail.com
[6] Departamento de Física, Instituto de Materiales y Nanotecnología, MALTA Consolider Team, Universidad de La Laguna, 38207 San Cristóbal de La Laguna, Spain; plrguez@ull.edu.es (P.R.-H.); amunoz@ull.edu.es (A.M.)
[7] ALBA-CELLS, MALTA Consolider Team, 08290 Cerdanyola del Valles (Barcelona), Catalonia, Spain; cpopescu@cells.es
* Correspondence: andreljp@ita.br (A.L.d.J.P.); fjmanjon@fis.upv.es (F.J.M.)

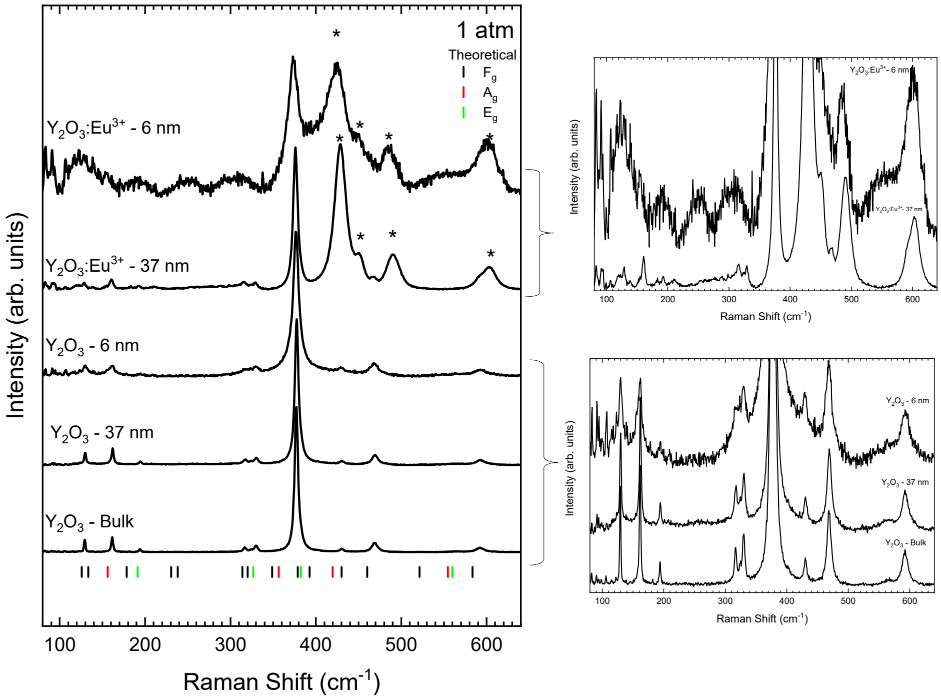

Figure S1. RS measurements of bulk Y_2O_3 and pure and Eu^{3+}-doped Y_2O_3 nanocrystals at ambient pressure (outside the DAC). Peaks marked with * are related to the luminescence of Eu^{3+} ions in the Y_2O_3 lattice.

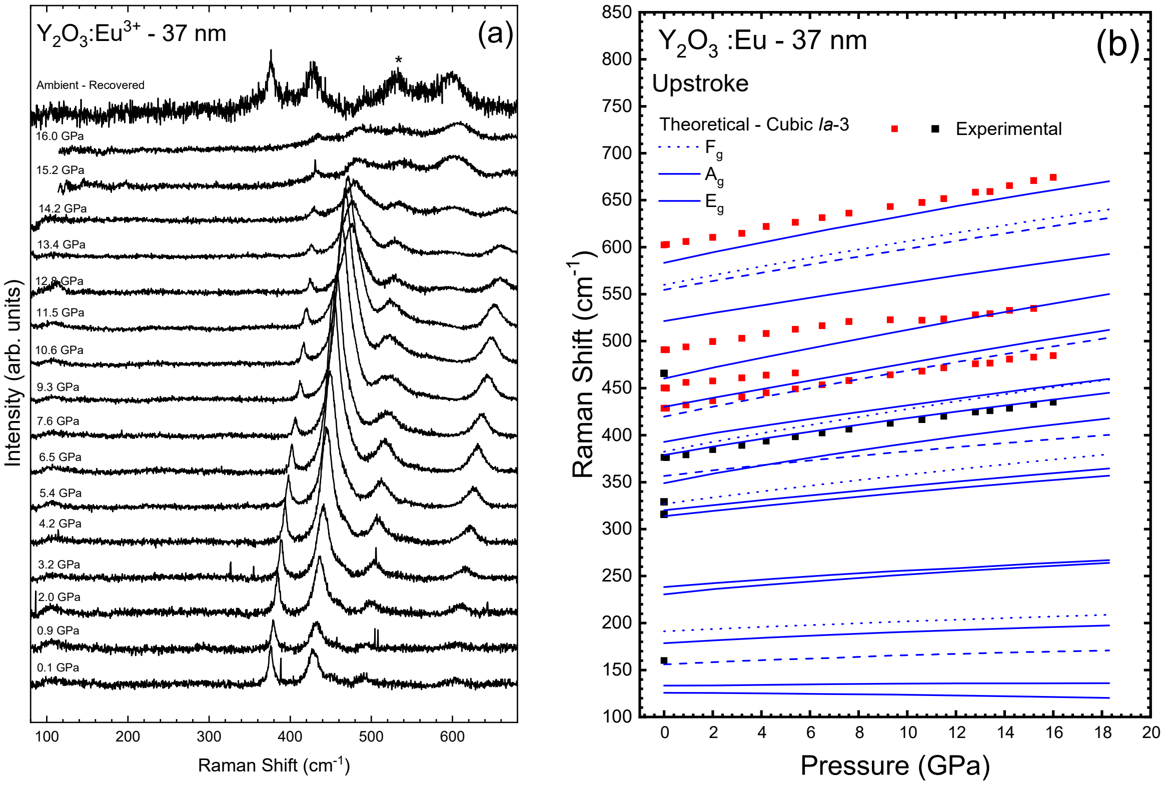

Figure S2. (a) Room-temperature Raman spectra of $Y_2O_3:Eu^{3+}$-37nm at selected pressures on upstroke. The upper spectrum is related to the recovered sample after decompression and the * indicates a peak unrelated to the initial phase. (b) Pressure dependence of the experimental (symbols) Raman-active frequencies of $Y_2O_3:Eu^{3+}$-37nm on upstroke. Blue lines represent the theoretical Raman-active frequencies of bulk C-type Y_2O_3. Red symbols represent peaks related to the Eu^{3+} luminescence.

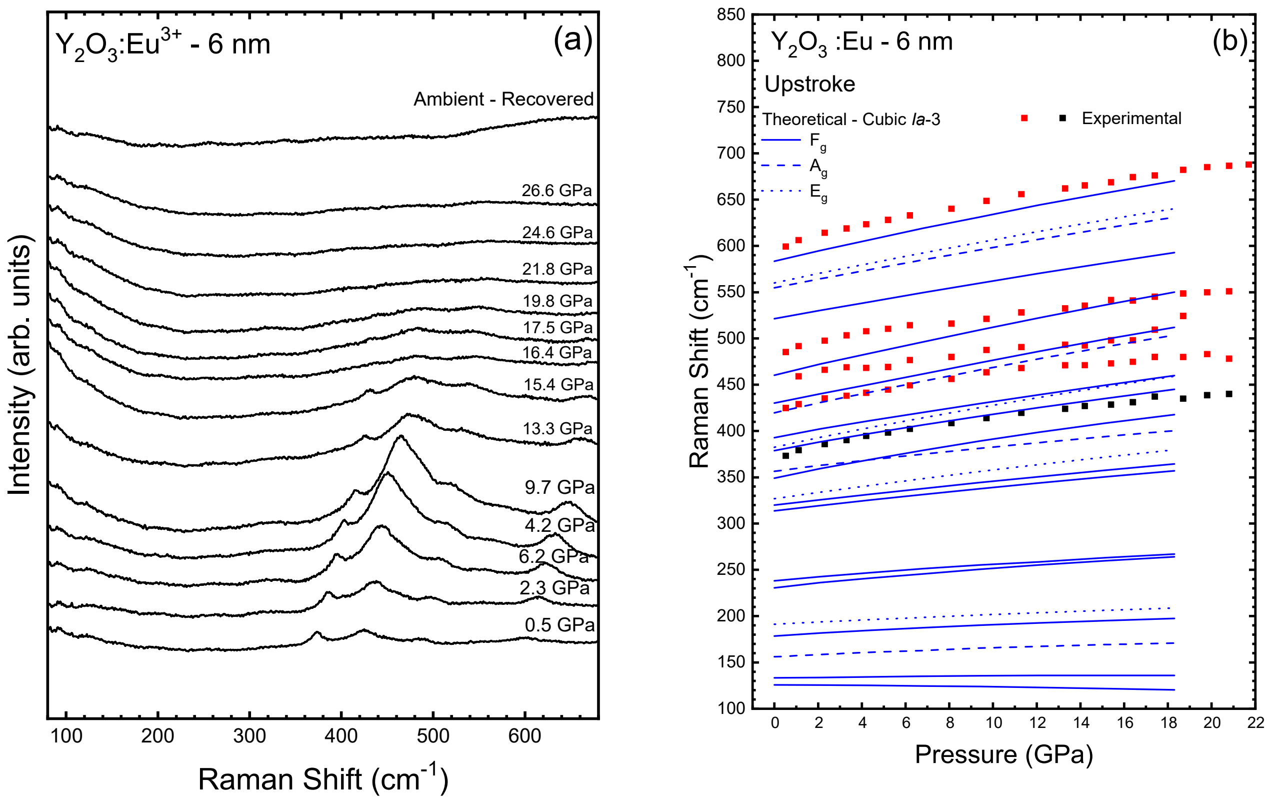

Figure S3. (a) Room-temperature Raman spectra of $Y_2O_3:Eu^{3+}$-6nm at selected pressures on upstroke. The upper spectrum is related to the recovered sample after decompression. (b) Pressure dependence of the experimental (symbols) Raman-active frequencies of $Y_2O_3:Eu^{3+}$-6nm on upstroke. Blue lines represent the theoretical Raman-active frequencies of bulk C-type Y_2O_3. Red symbols represent peaks related to the Eu^{3+} luminescence.

1.
